# The Choice of an Appropriate Information Dissimilarity Measure for Hierarchical Clustering of River Streamflow Time Series, Based on Calculated Lyapunov Exponent and Kolmogorov Measures

**DOI:** 10.3390/e21020215

**Published:** 2019-02-23

**Authors:** Dragutin T. Mihailović, Emilija Nikolić-Đorić, Slavica Malinović-Milićević, Vijay P. Singh, Anja Mihailović, Tatijana Stošić, Borko Stošić, Nusret Drešković

**Affiliations:** 1 Faculty of Agriculture, University of Novi Sad, Dositej Obradovic Sq. 8, 21000 Novi Sad, Serbia; 2 ACIMSI—Center for Meteorology and Environmental Modeling, University of Novi Sad, Dositej Obradovic Sq. 7, 21000 Novi Sad, Serbia; 3 Department of Biological and Agricultural Engineering and Zachry Department of Civil Engineering, Texas A&M University, College Station, TX 77843-2117, USA; 4 Departamento de Estatística e Informática, Universidade Federal Rural de Pernambuco, Rua Dom Manoel de Medeiros s/n, DoisIrmãos, 52171-900 Recife, Brazil; 5 Faculty of Sciences, Department of Geography, University of Sarajevo, Zmaj from Bosnia 33–35, 71000 Sarajevo, Bosnia and Herzegovina

**Keywords:** streamflow time series, Brazos River, average-linkage clustering hierarchical algorithm, K-means clustering, Kolmogorov complexity-based measures, largest Lyapunov exponent, Lyapunov time, Kolmogorov time, predictability of streamflow time series

## Abstract

The purpose of this paper was to choose an appropriate information dissimilarity measure for hierarchical clustering of daily streamflow discharge data, from twelve gauging stations on the Brazos River in Texas (USA), for the period 1989–2016. For that purpose, we selected and compared the average-linkage clustering hierarchical algorithm based on the compression-based dissimilarity measure (NCD), permutation distribution dissimilarity measure (PDDM), and Kolmogorov distance (KD). The algorithm was also compared with K-means clustering based on Kolmogorov complexity (KC), the highest value of Kolmogorov complexity spectrum (KCM), and the largest Lyapunov exponent (LLE). Using a dissimilarity matrix based on NCD, PDDM, and KD for daily streamflow, the agglomerative average-linkage hierarchical algorithm was applied. The key findings of this study are that: (i) The KD clustering algorithm is the most suitable among others; (ii) ANOVA analysis shows that there exist highly significant differences between mean values of four clusters, confirming that the choice of the number of clusters was suitably done; and (iii) from the clustering we found that the predictability of streamflow data of the Brazos River given by the Lyapunov time (LT), corrected for randomness by Kolmogorov time (KT) in days, lies in the interval from two to five days.

## 1. Introduction

Cluster analysis (also called clustering) is employed to identify the set of objects with similar characteristics or identify groups, and has a broad range of applications in science (e.g., biology, computational biology and bioinformatics, medicine, hydrology, geosciences, business and marketing, computer science, social science, and others). The analysis hypothesizes that the objects in the same group are more similar to each other than to those in other groups. The question; however, arises: What is the purpose of doing that? The purpose can be stated as to: (i) Identify the underlying structures in data; (ii) summarize behaviors or characteristics; (iii) assign new individuals to groups; and (iv) identify totally atypical objects [1,2,3]. Clusters are created by choosing variables that are either active or illustrative input variables. The active variables are often (but not always) numeric variables, while the illustrative variables are used for understanding the characteristics on which the clusters are based and, hence, for their interpretation. For grouping objects, a measure of nearness or proximity measure is needed. The closeness of objects can be measured by the degree of distance (a dissimilarity measure) or by the degree of association (a measure of similarity between groups). If two objects are more alike the dissimilarity measure decreases, while the similarity measure increases [4]. There are different methods for quantifying the similarity or dissimilarity measure and, hence, clustering, such as partitioning, hierarchical, fuzzy, density-based, and model-based.

This paper compares the average-linkage clustering hierarchical algorithm based on the compression-based dissimilarity measure (NCD), permutation distribution dissimilarity measure (PDDM), and Kolmogorov Distance (KD) for daily streamflow discharge data from twelve gauging stations on the Brazos River in Texas (USA), for the period 1989–2016. Each of the applied dissimilarity measures are distances and so they are non-negative, symmetric, and they satisfy triangle inequality. The algorithm is also compared with K-means clustering based on Kolmogorov complexity (KC), the highest value of Kolmogorov complexity spectrum (KCM), and the largest Lyapunov exponent (LLE). This analysis should reveal the differences in the selected clusters for streamflow. The combination of such a clustering method and information measures can be a useful tool for time series modeling, interpolation, and data mining; delineation of homogeneous hydrometeorological regions; catchment classification; regionalization of catchments for flood frequency analysis and prediction in ungauged basins; and hydrological modeling, flood forecasting, and estimation of predictive uncertainty [5,6,7,8,9]; among others. In the agglomerative hierarchical approach, we define each data point as a cluster and combine existing clusters at each step. Depending on the definition of the distance between two clusters, there exist different agglomerative techniques. The most frequently applied techniques are: the single-linkage, average-linkage, complete-linkage, centroid-linkage, Ward’s method, and McQuitty linkage [4]. In practice, the linkage function is usually more important than the distance function itself [10]. Each of these linkage functions can give different results when used on the same data set. According to [11] and [4] the single-link method is the most versatile algorithm and is sensitive to data errors and chaining. The complete-linkage method is strongly affected by outliers, as it is based on maximum distances. Centroid- and average-linkage are affected by outliers but less than the complete-linkage method. Comparing the single-link, complete-link, average-link, centroid, and Ward methods, [12] found the average-link method to be the preferred method. As the focus of our study is information dissimilarity measures, the presentation of results is restricted to the average-linkage function. The other linkage functions were also considered. In the case when the dissimilarity measure is Kolmogorov complexity distance, the obtained grouping is the same for average-linkage, complete-linkage, centroid method, Ward’s method, and McQuitty linkage, either for three or four clusters. If the compression-based dissimilarity measure or permutation distribution dissimilarity measure are applied, grouping in clusters depends on the choice of linkage function.

The paper is organized as follows. Section 2 describes the selected clustering hierarchical algorithms (compression-based dissimilarity measure, permutation distribution dissimilarity measure, and Kolmogorov distance). Section 3 provides information on streamflow data and gauging locations. Section 4 presents the results obtained, discussing the average-linkage clustering hierarchical algorithm with respect to three dissimilarity measures, and K-means clustering based on the above mentioned information measures. The concluding remarks are given in Section 5.

## 2. Data and Computations

### 2.1. Data and Gauging Locations 

**Figure 1:**
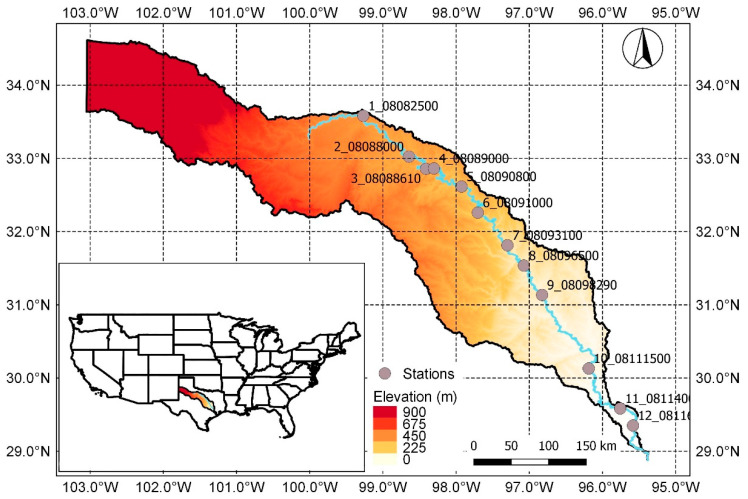
Geographical locations of the gauging stations on the Brazos River used in this study.

Daily streamflow values were obtained from the National Water Information System: Web Interface at https://waterdata.usgs.gov/nwis, for the Brazos River in Texas (USA), which has a drainage area of approximately 118,000 km^2^, extending from eastern New Mexico to more than 1000 km southeast to the Gulf of Mexico. Daily streamflow observations from 12 USGS stream gauges on the mainstream were obtained for the period from 1989 to 2016, when simultaneous data for all the stations were available. The geographical locations of gauging stations are depicted in Figure 1, and basic statistics of data are given in Table 1.

Daily discharge data were standardized (i.e., for each calendar day “*i*” means discharge 〈$x_{i}$〉 and standard deviation ${\mathrm{SD}}_{i}$, over the year, “*j*”, were computed and then the standardized discharge on day “*i*” in year “*j*” was calculated as $y_{i,j}=\left(x_{i,j}-\langle x_{i}\rangle \right)/{\mathrm{SD}}_{i}$ [13]. This procedure removes any seasonal effects.

### 2.2. Basic Descriptive Statistics 

**Figure 2:**
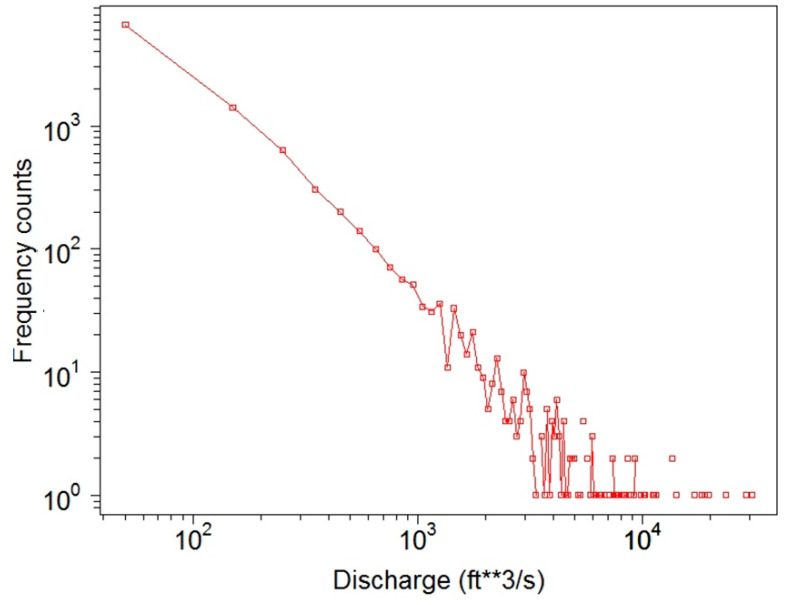
Frequency counts of daily discharge data for the USGS 08082500 Brazos River station at Seymour, Texas (USA) for the period 1989–2016.

Basic descriptive statistics of daily discharge data of the gauging stations are summarized in Table 1, where for each station the mean, median, minimum, maximum, interquartile range (IQR), and standard deviation (${\mathrm{SD}}_{i}$) are shown. It is seen from Table 1 that the differences between the maximum and the mean values are in the range of roughly 10 to 40 standard deviations, strongly positively skewed, indicating a power law behavior. Indeed, frequency counts for the USGS 08082500 Brazos River station at Seymour, Texas (USA), displayed in Figure 2 on a log–log scale, demonstrated a power law distribution, with similar behavior also observed at all other stations.

## 3. Method

In this section, we describe the selected dissimilarity measures (compression-based dissimilarity measure, permutation distribution dissimilarity measure, and Kolmogorov distance), used in the average-linkage clustering hierarchical algorithm, which was applied to streamflow data measured from 12 gauging locations on the Brazos River in Texas (USA). We also briefly consider three information measures (the largest Lyapunov exponent, Kolmogorov complexity, and the highest value of the Kolmogorov spectrum) used for K-means clustering based on these information measures.

### 3.1. Choice of Measures for Characterization of Streamflow for Clustering

Cluster analysis of gauged streamflow records into regions is an important tool for the characterization of hydrologic systems. To that end, the distance between two gauge stations, i and j, is frequently measured by the Euclidean distance $d_{ij}$ (ED) [14,15,16], which is expressed as:

(1) $$d_{ij}=\sqrt{\overset{p}{\underset{k=1}{\sum }}{\left(x_{ik}-y_{jk}\right)}^{2},}$$

where $\left\{x_{ik}\right\}$ and $\left\{y_{jk}\right\}\;k=1,\ldots ,p$ are the streamflow values at the stations, while p is the period which can be daily, monthly, seasonal, or annual. Although there are many other distance metrics, this distance is frequently used as a dissimilarity measure in the clustering algorithms. Gong and Richman [15] showed that the majority of investigators (about 85%) applied this measure in their studies [5]. Despite the popularity of the ED measure in streamflow clustering, it has a drawback in that it assumes that the sample points are distributed about the sample mean in a spherical manner. If the distribution happens to be decisively non-spherical, for example ellipsoidal, then we would expect the probability of a “test point” belonging to the set to depend not only on the distance from the sample mean but also on the direction. The dynamic time warping (DTW) is a more general algorithm, based on ED, that enables the finding of the best alignment between time series that may have different lengths and/or local distortions [17,18]. Besides a shape-based measure, the dissimilarity of time series may be measured by comparing the features extracted from the original time series, such as autocorrelations, cross-correlations, spectral features, wavelet coefficients, and information measures, or by model approach [19]. Among many information measures we have tested in this study, we selected three measures for the characterization of streamflow: compression based dissimilarity measure (NCD), permutation distribution dissimilarity measure (PDDM), and Kolmogorov distance (KD).

The normalized compression distance (NCD) provides a computable version of the normalized information distance (NID). It has been recommended for application in bioinformatics, music clustering, linguistics, plagiarism detection, image similarity, question answering, and many other fields [20]. This measure has a broad application in the clustering of heterogeneous data. Therefore, it can be used for clustering streamflow, for example, in optimizing streamflow monitoring networks on the basis of daily streamflow data.

Permutation distribution dissimilarity measure (PDDM) is a complexity-based approach to clustering time series. The dissimilarity of time series is formalized as the squared Hellinger distance between the permutation distributions of embedded time series [21]. This method has not been used in hydrology that often in the past. However, recently some authors used it for multiscale parameter regionalization implemented within a spatially-distributed mesoscale hydrologic model, clustering streamflow time series for regional classification, and establishing relationships between the regionalization and streamflow indices [22,23,24].

The Kolmogorov complexity distance (KD) has become an important tool in a wide variety of applications [25]. It has also been applied in hydrology in scaling problems, since the heterogeneity of catchments and the variability of hydrological processes make scaling (which is performed either in a deterministic or a stochastic framework) so difficult [26]. In this study, we used this measure for clustering streamflow. To our knowledge, this measure for this purpose has not been applied yet.

### 3.2. Normalized Compression Distance

A normalized information distance (NID) between two objects (time series, images, texts) x and y is given by:

(2) $$\mathrm{NID}=\frac{max\left\{K\left(x|y\right),K\left(y|x\right)\right\}}{max\left\{K\left(x\right),K\left(y\right)\right\}},$$

where the conditional Kolmogorov complexity K(x|y) of x, given y, is the length of the shortest program producing x when y is given as an auxiliary input in the program. The NID is theoretically appealing, but not practical, since it cannot be computed. In this subsection, we consider the normalized compression distance (NCD), an efficiently computable, and thus practically applicable, form of the normalized information distance. One approach for computing NID is approximating Kolmogorov complexity by the length of the compressed objects obtained from some data compressors (gzip, bzip2, xz). Using the approximation K(x|y)≈K(xy)−K(y), the normalized compression distance (NCD) is defined as:

(3) $$\mathrm{NCD}=\frac{C\left(xy\right)-min\left\{C\left(x\right),C\left(y\right)\right\}}{max\left\{C\left(x\right),C\left(y\right)\right\}},$$

where C is a chosen data compressor, and C(xy) is the size in bytes of the series x and y concatenated. The function NCD from the R 3.5.1 package TSclust, applied in the calculation, selects the best compression algorithm separately for x,y and concatenated xy [27]. 

Sometimes, for simplicity, it is advisable to present calculations of the clustering matrix in the form of pseudocode, which is a detailed yet readable description of what a computer program or algorithm must do, expressed in a formally-styled natural language, rather than in a programming language. The pseudocode for calculating the NCD has the following steps:

1. Select data compressor C among available compressors (gzip, bzip2, xz).
2. Set the number of time series compressed by the chosen compressor C to *N*.
3. Set all elements of clustering matrix $M^{C}\left(N,N\right)$ to zero.
4. Calculate Kolmogorov complexity by the length of the compressed time series obtained from some data compressors C(x), C(y).
5. Calculate C(xy) which is the size in bytes of the time series x and y concatenated.
6. Find the lower value of {C(x),C(y)}.
7. Find the higher value of {C(x),C(y)}.
8. Calculate the normalized compressed distance (NCD) given by Equation (3).
9. Set the calculated value into $M^{C}\left(i,j\right)$ i=1, N−1; j=i+1, N.

### 3.3. Permutation Distribution Dissimilarity Measure

Permutation distribution dissimilarity measure (*PDDM*) is based on the distance of distributions of permutations [28]. On the basis of given time series $\left\{x_{t}\right\},\;t=1,\ldots ,N$, the m-dimensional embedding with time delay t is $X_{m}^{\prime }=\left\{\left(x_{i},\;x_{i+t},x_{i+2t},\ldots ,x_{i+\left(m-1\right)t}\right),\;i=1,\ldots ,N-\left(m-1\right)t\right\}$. For each $X_{m}^{\prime }$ permutation $\prod \left(X_{m}^{\prime }\right)$ obtained by sorting $X_{m}^{\prime }$ in the ascending order is recorded, and the distribution of permutations is denoted by $P\left(x_{t}\right)$. The dissimilarity between two time series is measured by the dissimilarity of their permutation distributions. One approach is based on Kullback-Leibler (KL) divergence (relative Shannon entropy). Taylor approximation of KL divergence is the squared Helling distance of discrete probability distributions $P=\left(p_{1},p_{2},\ldots ,p_{n}\right)$ and $Q=\left(q,q_{2},\ldots ,q_{n}\right)$: $D\left(P,Q\right)=\frac{1}{\sqrt{2}}\cdot {\left\|\sqrt{P}-\sqrt{Q}\right\|}_{2}^{2}$, where ${\left\|\;\right\|}_{2}$ is the Euclidean norm. The pseudocode for calculating the PDDM has the steps:

1. Set all elements of clustering matrix $M^{C}\left(N,N\right)$ to zero.
2. Use time series $\left\{x_{t}\right\},t=1,\ldots ,N$
3. For given time series, the m-dimensional embedding with time delay t is
  $X_{m}^{\prime }=\left\{\left(x_{i},\;x_{i+t},x_{i+2t},\ldots ,x_{i+\left(m-1\right)t}\right),\;i=1,\ldots ,N-\left(m-1\right)t\right\}.$
4. Sort $x_{m}^{\prime }$ in the ascending order to get permutation $\prod \left(X_{m}^{\prime }\right)$ for each $x_{m}^{\prime }.$
5. Obtain the distribution of permutations $P\left(x_{t}\right).$
6. Steps 2–5 for time series $\left\{y_{t}\right\},t=1,\ldots ,N.$
7. Calculate distance $D\left(P,Q\right)=\frac{1}{\sqrt{2}}\cdot {\left\|\sqrt{P}-\sqrt{Q}\right\|}_{2}^{2}$, where *P* and *Q* are discrete probability distributions.
8. Set calculated value of D(P,Q) into $M^{C}\left(N,N\right)\;i=1,\;N-1;\;j=i+1,\;N$.

### 3.4. Kolmogorov Complexity Distance (KD)

Kolmogorov complexity distance is defined using the conditional complexity as:

(4) $$\mathrm{KD}=\left\{\frac{K\left(x|y\right)-K\left(y|y\right)}{K\left(y|y\right)}\right\}+\left\{\frac{K\left(y|x\right)-K\left(x|x\right)}{K\left(x|x\right)}\right\}.$$

Since

K(x|y)≈K(xy)−K(y),
K(y|x)≈K(yx)−K(x),
K(x|x)≈K(xx)−K(x)

We get

(5) $${\mathrm{KD}}_{xy}=\left\{\frac{\left[K\left(xy\right)-K\left(y\right)\right]-\left[K\left(yy\right)-K\left(y\right)\right]}{\left[K\left(yy\right)-K\left(y\right)\right]}\right\}+\left\{\frac{\left[K\left(yx\right)-K\left(x\right)\right]-\left[K\left(xx\right)-K\left(x\right)\right]}{\left[K\left(xx\right)-K\left(x\right)\right]}\right\}.$$

While KD, as given by (5), is a non-negative and symmetric quantity, it does not in general satisfy the triangle inequality. Therefore, after calculating KD distance matrix using (5), all pairs are checked: if for a given pair of objects x,y it turns that ${\mathrm{KD}}_{xy}>min_{z}\left[{\mathrm{KD}}_{xz}+{\mathrm{KD}}_{zy}\right]$, then the distance is set to ${\mathrm{KD}}_{xy}=min_{z}\left[{\mathrm{KD}}_{xz}+{\mathrm{KD}}_{zy}\right]$, and all the pairs are checked anew. The true distance is computed by iterating this procedure until for all x,y and z the triangle inequality is satisfied ${\mathrm{KD}}_{xy}\leq {\mathrm{KD}}_{xy}+{\mathrm{KD}}_{zy}$.

When the matrix $D_{\left(mxm\right)}$ of distances of all pairs of p objects is obtained by any of the three selected information distances, hierarchical clustering is performed. The average linkage clustering defines the distance between any two clusters to be the average of distances of all pairs of objects from any member of one cluster from any member of the other cluster. The pseudocode for KD has the following steps:

1. Set all elements of clustering matrix $M^{C}\left(N,N\right)$ to zero.
2. Calculate distances ${\mathrm{KD}}_{xy},{\mathrm{KD}}_{xz},{\;\mathrm{and}\;\mathrm{KD}}_{zy}$ using Equation (5).
3. Check for all pairs: If for a given pair of time series x,*y* it turns that ${\mathrm{KD}}_{xy}>min_{z}\left[{\;\mathrm{KD}}_{xy}+{\;\mathrm{KD}}_{zy}\right]$ then the distance is set to ${\;\mathrm{KD}}_{xy}=min_{z}\left[{\;\mathrm{KD}}_{xy}+{\;\mathrm{KD}}_{zy}\right].$
4. The true distance is computed by iterating this procedure until for all x,y and z the triangle inequality is satisfied ${\mathrm{KD}}_{xy}\leq {\;\mathrm{KD}}_{xy}+{\;\mathrm{KD}}_{zy}$.
5. Set the calculated value of ${\mathrm{KD}}_{xy}$ into $M^{C}\left(i,j\right)\;i=1,\;N-1;\;j=i+1,\;N$.

### 3.5. Calculation of Largest Lyapunov Exponent and Kolmogorov Measures

Because the rate of separation can be different for different orientations of the initial separation vector, there is a spectrum of Lyapunov exponents whose largest value is commonly assigned as LLE. A positive value of this exponent is usually taken as an indication that the system is chaotic. In this study, we obtained the LLE for the standardized daily discharge time series by applying the Rosenstein algorithm [29], which was implemented in MATLAB program [30]. This algorithm is fast, easy to apply, and robust to changes in the embedding dimension, reconstruction delay, length of time series, and noise level. The applied MATLAB program calculates the proper embedding dimension and reconstruction delay. The value of embedding dimension is selected by the FNN (false nearest neighbors) method or the symplectic geometry method in the case of high noisy data [31,32]. The Kolmogorov complexity and its derivates (the Kolmogorov spectrum and its highest value) are calculated using the Lempel Ziv algorithm, which is widely described in [33].

## 4. Results and Discussion

### 4.1. Selection of Information Measures for K-Means Clustering of Daily Streamflow 

The question arises: How to select the information measures for K-means clustering? We selected three measures (i.e., the largest Lyapunov exponent (LLE), Kolmogorov complexity (KC), and the highest value of the Kolmogorov complexity (KCM). This choice was made for the following reasons.

#### 4.1.1. General Features

(i) The Brazos River course has a large interval of mean daily streamflow values, which ranged from 223.5 [Seymour station (1_08082500)] to 8851.4 m^3^/s [Roshanor station (12_08116650)], as seen in Table 1.

(ii) There are many factors, both natural (runoff from rainfall and snowmelt; evaporation from soil and surface-water bodies; transpiration by vegetation; ground-water discharge from aquifers; ground-water recharge from surface-water bodies; sedimentation of lakes and wetlands, etc.) and human-induced (surface-water withdrawals and transbasin diversions; river-flow regulation for hydropower and navigation; dams; construction, removal, and sedimentation of reservoirs and storm water detention ponds; stream channelization and levee construction; drainage or restoration of wetlands; land-use changes, such as urbanization, that alter rates of erosion, infiltration, overland flow, and evapotranspiration; wastewater outfalls; and irrigation wastewater return flow), that cause continuous changes in streamflow time series and; therefore, in its nonlinearity and complexity of the Brazos River and its drainage basin. For example, a huge human intervention was the Morris Sheppard Hydroelectric Power Plant at Morris Sheppard Dam (Possum Kingdom Reservoir) on the Brazos River in Palo Pinto County, built in the period 1938–1941 (11 miles southwest of Graford and 18 miles northeast from Mineral Wells). Currently, “USGS station 3_08088610 (Brazos River near Graford, Texas) is located approximately 1.25 miles downstream of Possum Kingdom Reservoir. As such, this site is largely influenced by regulation. This gage was established to monitor outflow from Possum Kingdom Reservoir. The gage was initially located farther upstream, closer to the outflow from the reservoir. In 1995, the gage was moved downstream to the current location” [34]. Another regulation on the Brazos River was the Aquilla Lake, which is an artificial lake in Hill County. The dam for this regulation was constructed by the U.S. Army Corps of Engineers. This dam is part of the overall flood control project in the Brazos River basin (station 7_08093100).

(iii) Because streamflow processes are unavoidably influenced by measurement at gauging stations (including uncertainties in the single determination of river discharge [35]) and dynamical noises that increase Lyapunov exponents under the influence of noise, then these factors were taken into considerations.

#### 4.1.2. Largest Lyapunov Exponent (LLE)

The perpetual debate over whether hydrological systems are deterministic or stochastic has been taken to a new level by controversial applications of nonlinear dynamics tools. Lyapunov exponents, perhaps the most informative invariants of a complex dynamical process, are also among the most difficult to determine from experimental data, although when using embedding theory to build chaotic attractors in a reconstruction space, extra “spurious” Lyapunov exponents arise that are not Lyapunov exponents of the original system [36,37]. Some hydrologists have discussed the difficulties and uncertainties in discerning between low-dimensional chaotic and stochastic systems using Lyapunov exponents and correlation dimension measures [38,39,40]. Thus, for the analysis of weak chaos, generating two phenomena from the normal functioning of the same system, the LLE has to be utilized carefully. In real physical systems, the structure of chaos is more complex than in truly random processes [41]. Systems with chaotic dynamics usually contain islands of stability. Accordingly, if the larger is the covering factor of the islands of stability, the weaker is the chaos. Intermittency is also one of the manifestations of the weak chaos [42]. Intermittent behavior is frequently observed in fluid flows that are turbulent or near the transition to turbulence. There are also numerous examples of weak chaos in hydrology. A further quantitative measure of weak chaos is the low dimension (close to 2) of the strange attractors characterizing their dynamics [43]. Wu et al. [44] offered another quantification of weak chaos when LLE is less than 0.1. They noted “If emergence is unapparent, the emergent time may be misjudged, which may lead to erroneous calculation of LLE. However, the LLE at a longer time is still positive, which manifests that chaos exists”.

Table 2 shows the LLE of standardized daily discharge data, indicating that station 3_08088610 (Graford) and1_08082500 (Seymour) had the highest value of LLE (0.394 and 0.158, respectively), while all other stations had values in the interval (0.018, 0.061) (i.e., in the region of weak chaos). Using LLE as an indicator, [45] established the presence of low chaos in the daily streamflow of the Kizilirmak River (Iran), with a positive value of LLE (0.0061). Similarly, in forecasting of daily streamflow of Xijiang River (China), [46] reported that LLE was 0.1604. The streamflow of station 1_08082500 (Seymour) had a value of 0.158 for LLE, which is approximately 2.6–8.8 times larger than for other stations. Following the criterion by [44], the streamflow, measured at this station, exhibited high chaotic behavior. In our opinion it comes from several reasons: (i) Uncertainties as a result of errors in the field determination of discharge [35,47]. In a project report, Ward [48] gave a judgment of the quality of field in the measurement of discharge for eleven selected Texas stream gauges (for the period 1987–2011), presumably based on the conditions of field work. He reported that the uncertainties, as relative standard errors (RSE), for discharge measurements for all stations, in general, were considerably larger than recommended by [47]. Surprisingly, 1_08082500 (Seymour) had the highest values of RSE (relative standard error)—188%—pointing to a high level of variability and a potential source of high nonlinearity (higher values of LLE) and randomness. (ii) The Seymour gauging station posted an extremely high sediment yield, while the next downstream gage (South Bend) showed a considerable decrease. In fact, sediment yields at Seymour (1220 t km^−2^ yr^−1^) were the highest among all the gauging stations on the Brazos River, whose average annual suspended-sediment yield is generally considered the highest of all rivers in the state of Texas [49]. Having in mind that a nonlinear relationship is inherent in the streamflow–suspended sediment relationship [50], the higher value of LLE could be attributed to this phenomenon. The highest value of LLE (0.394) for 3_08088610 (Graford) station is a result of changed river streamflow dynamics because of the Sheppard Hydroelectric Power Plant at Morris Sheppard Dam (Possum Kingdom Reservoir) that is built on the Brazos River in Palo Pinto County. More details about the change in the nonlinearity and randomness of streamflow for this gauge station can be found in [51].

#### 4.1.3. Kolmogorov Complexity (KC) and the Highest Value of Kolmogorov Complexity Spectrum (KCM)

The Kolmogorov complexity measures applied in this paper sheds additional light on the complex behavior of streamflow. The values of KC and KCM of standardized daily discharge data are shown in Table 2, which shows that the KC values for all daily streamflow time series were relatively small, ranging in the interval from 0.200 to 0.474. Similar behavior was observed for KCM, having values in the intervals from 0.252 to 0.682, which is expected for lowland rivers in contrast to mountain rivers, whose KC values can be up to 0.98 [51,52]. From Table 2 it is seen that there were three peaks for KC: 0.474, 0.352, and 0.316 for stations 3_08088610 (Graford), 7_08093100 (Aquilla), and 9_08098290 (Highbank), respectively. The highest value of KC (3_08088610) was a result of the human activity (i.e., the building of a hydroelectric power plant changing the streamflow dynamics; see the previous subsection). It would be interesting to clarify the appearance of peaks in KC values for 7_08093100 (Aquilla) and 9_08098290 (Highbank) stations. Both stations had low values of LLE (0.055 and 0.061), which belong to the domain of weak chaos (i.e., they are very close to zero). We now had a situation of the occurrence of stochastic behavior (high randomness), although LLE indicated a stable state. Vilela Mendes [53] explained this situation in the following way. The idea is that the dynamics is simple to describe in law, but not that it has simple orbits. In short, a dynamical law with small sophistication but capable of generating orbits of high Kolmogorov complexity. According to [54], “sophistication” is defined as the size of the projectable part of the string’s minimal description and formalizes the amount of planning which went into the construction of the string. Note that an additional source of the occurrence of peak for 7_08093100 (Aquilla) station was because of human intervention (i.e., the presence of the Aquilla dam).

### 4.2. Hierarchical Clustering of Daily Streamflow

**Figure 3:**
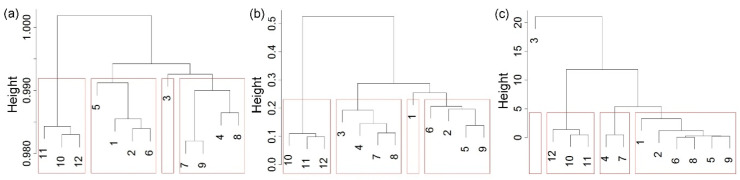
Dendrogram for hierarchical clustering of daily streamflow based on applied dissimilarity measure: (**a**) Compression-based dissimilarity measure; (**b**) permutation distribution dissimilarity measure; and (**c**) Kolmogorov complexity distance.

Starting with a dissimilarity matrix based on the compression-based dissimilarity measure (NCD), permutation distribution dissimilarity measure (PDDM), and Kolmogorov distance (KD) for daily streamflow discharge data from twelve gauging stations on the Brazos River in Texas (USA), for the period 1989–2016, the agglomerative average-linkage hierarchical algorithm was applied. This algorithm consists of a series of successive fusions of the objects into groups culminating in the stage where all objects are in one group. At any stage in the procedure, two objects or groups of objects which are the closest are fused together. The average-linkage clustering defines a distance between any two groups of objects (clusters) to be the average of distances of all pairs of objects, from any member of one cluster to any member of the other cluster. The tree diagram (dendogram) gives the stages in the aggregation of gauging stations in clusters (Figure 3). The vertical axis is used to indicate the distances at which the joining occurs.

**Figure 4:**
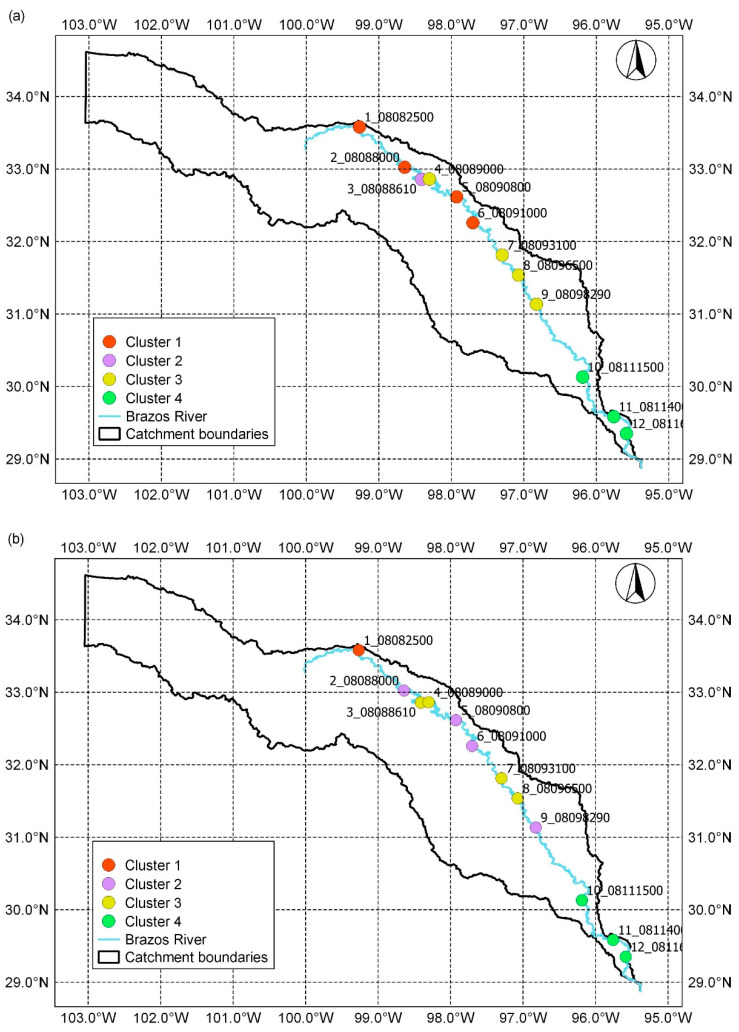

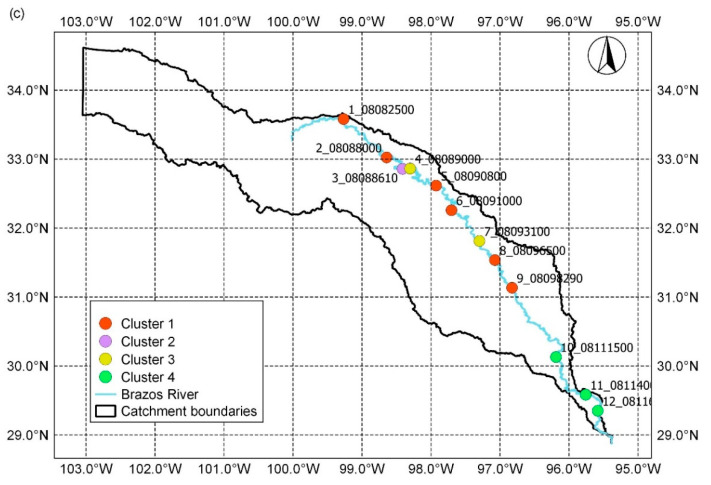
Map of hierarchical clustering of daily streamflow based on the applied dissimilarity measure: (**a**) Compression-based dissimilarity measure; (**b**) permutation distribution dissimilarity measure; and (**c**) Kolmogorov complexity distance.

The dendrogram gives the indication that the stations may be grouped either in three or four clusters. For comparison with the results of the latter analysis, we chose four clusters. The results of grouping were visualized on maps of the geographical locations of gauging stations on the Brazos River used in this study (Figure 4). If the compression-based dissimilarity measure was applied, the stations were distributed as: (i) Cluster 1 (1_08082500, 2_08088000, 5_08090800, and 6_08091000); Cluster 2 (3_08088610); Cluster 3 (4_08089000, 7_08093100, 8_08096500, and 9_08098290); and Cluster 4 (10_08111500, 11_08114000, and 12_08116650). The hierarchical clustering based on permutation distribution dissimilarity measure gave: (i) Cluster 1 (1_08082500); Cluster 2 (2_08088000, 5_08090800, 6_08091000, and 9_08098290); Cluster 3 (3_08088610, 4_08089000, 7_08093100; and 8_08096500), and Cluster 4 (10_08111500, 11_08114000, and 12_08116650). In the case when the dissimilarity measure was Kolmogorov complexity distance, the obtained grouping was: (i) Cluster 1 (1_08082500, 2_08088000, 5_08090800, 6_08091000, 8_08096500, and 9_08098290), Cluster 2 (3_08088610); Cluster 3 (4_08089000 and 7_08093100); and Cluster 4 (10_08111500, 11_08114000, and 12_08116650). It may be noted that, in all cases, stations 10_08111500, 11_08114000, and 12_08116650 belonged to the same cluster. 

In computer science, the computational complexity of an algorithm is the amount of resources required for running it. The computational complexity of a problem is the minimum of the complexities of all possible algorithms for this problem (including the unknown algorithms). Here we shortly present a comparative analysis of the computational complexity of all the three used algorithms. We have chosen for criterion the computational cost, which depends not only on the size of the dataset, but also on the complexity of many other aspects. For comparison we used three times. The “user time” (UT) is the CPU time charged for the execution of user instructions of the calling process. The “system time” (ST) is the CPU time charged for execution by the system on behalf of the calling process. The first two entries are the total user and system CPU times of the current R (language) process and any child processes on which it has waited, and the third entry is the “real elapsed time” (RET) since the process was started.

For 12 time series, each with a size of 9968 samples, we obtained the following results: (1) NCD (UT = 36.97; ST = 1.67; RET = 52.11); (2) PDDM (UT = 0.17; ST = 0.04; RET = 0.20); and (3) KD (UT = 19.13; ST = 1.13; RET = 29.24).

The focus of this paper is to suggest a suitable information dissimilarity measure for hierarchical clustering of river streamflow time series, but without going down into detailed aspects in comparison of the clustering algorithms we have used. However, in discussion, we cannot avoid the aspect of data size in time series clustering. Shortly, we will do it in the following way. Time series clustering is a very effective approach in discovering valuable information in various systems. However, focusing on the efficiency and scalability of these algorithms to deal with time series data has come at the expense of losing the usability and effectiveness of clustering. Aghabozorgi and Teh [55] proposed a method, which was compared with different algorithms and various datasets of dissimilar length, showing that this method outperformed other conventional clustering algorithms. They emphasized that the user does not require very low-resolution time series for clustering of large datasets; instead, the clustering can be applied on smaller sets of high dimension time series by the prototyping process. That is, the cost of using representatives is much less than the dimension reduction in terms of accuracy.

### 4.3. K-Means Clustering of Daily Streamflow

**Figure 5:**
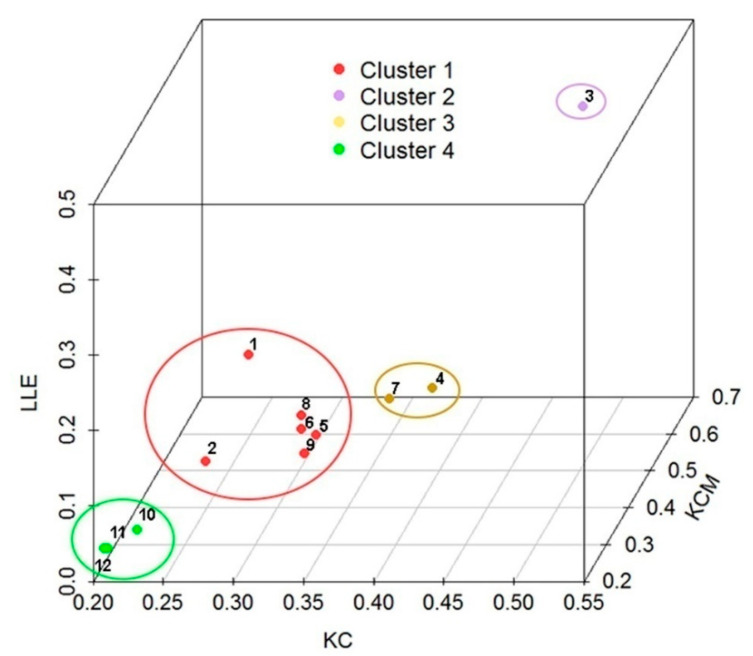
3D scatter plot specified by the vectors KC, KCM, and LLE.

**Figure 6:**
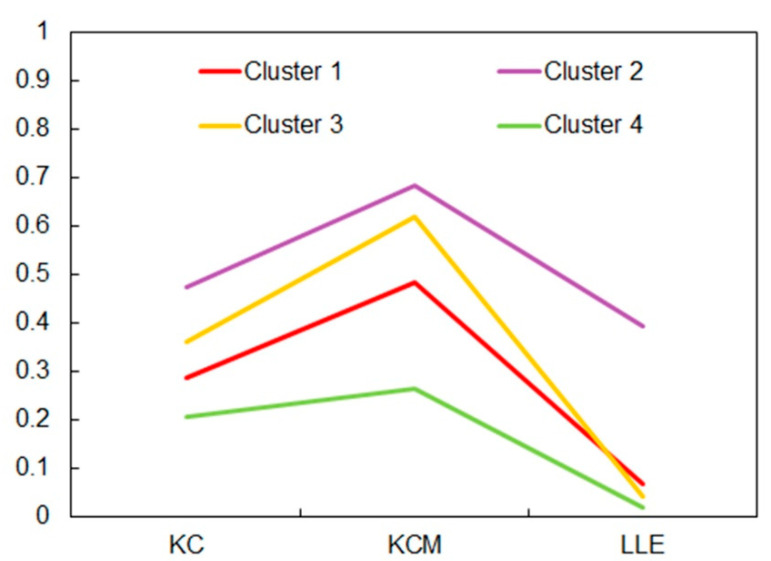
Plot of means for all clusters.

The first step in the K-means clustering is the determination of the number of clusters K. The 3D scatter plot of points (KC*_i_,* KCM*_i_,* LLE*_i_*) (*i* = 1, …, 12), which were calculated on the basis of daily discharge data recorded during the period 1989–2016 at twelve gauging stations on the Brazos River in Texas, suggested that K = 4, as shown by the 3D scatter plot in Figure 5. From this figure it can be seen that clustering closely followed the aforementioned discussion about the choice of information measures for K-means clustering. The K-means algorithm consists of repeating three steps until convergence: (i) Determining the centroid coordinate; (ii) determining the distance of each object to the centroids; and (iii) grouping the objects based on minimum distance to their closest cluster center, according to the Euclidean distance function. Any random object may be taken as the initial centroid. We applied the program STATISTICA 13.2 for K-means clustering. The stations were distributed in the following ways: (i) Cluster 1 (1_08082500, 2_08088000, 5_08090800, 6_08091000, 8_08096500, and 9_08098290); Cluster 2 (3_08088610); Cluster 3 (4_08089000 and 7_08093100); and Cluster 4 (10_08111500, 11_08114000, and 12_08116650). Table 3 and Figure 6 show the centroids of the clusters. Cluster 2 had the highest values, while cluster 4 had the lowest values of the mean values of all considered information measures.

On the basis of the analysis of variance (ANOVA) results (Table 4), it could be concluded that there existed highly significant differences between mean values of four clusters, which confirms that the choice of the number of clusters was correctly done.

For the end of discussion, let us consider the question about predictability of streamflow, seen through the light of the aforementioned consideration of clustering the streamflow time series. The Lyapunov exponent relates to the predictability of measured time series, which includes deterministic chaos as an inherent component. Model predictability is here understood as the degree to which a correct prediction of a system’s state can be made, either qualitatively or quantitatively. In stochastic analysis, a random process is considered predictable if it is possible to infer the next state from previous observations. In many models; however, randomness is a phenomenon which “spoils” predictability [51]. Deterministic chaos does not mechanically denote total predictability, but means that at least it improves the prognostic power. In contrast, stochastic trajectories cannot be projected into future. If LLE>1 then streamflow is not chaotic, but is rather stochastic, and predictions cannot be based on chaos theory. However, if 0<LLE<1 it indicates the existence of chaos in streamflow. In that case, one can compute the approximate time (often called Lyapunov time (LT)) limit for which accurate prediction for a chaotic system is a function of LLE. It designates a period when a certain process (physical, mechanical, hydrological, quantum, or even biological) moves beyond the bounds of precise (or probabilistic) predictability and enters a chaotic mode. According to [56], that time can be calculated as $\Delta t_{lyap}=1/\mathrm{LLE}$. If LLE→0, implying that $\Delta t_{lyap}\to \infty$, then long-term accurate predictions are possible. However, many streamflow time series are highly complex. Therefore, Δ*t_lyap_* can be corrected for randomness in the following way. Similar to $\Delta t_{lyap},$ we can introduce a randomness time $\Delta t_{rand}=1/\mathrm{KC}$ (in time units, second, hour, or day). Henceforth, we shall denote this quantity Kolmogorov time (KT), as it quantifies the time span beyond which randomness significantly influences predictability. Then, the Lyapunov time corrected for randomness is defined as $\left[0,\Delta t_{lyap}\right]\cap \left[0,\Delta t_{rand}\right]$. It can be stated that the KT designates the size of the time window within time series where complexity remains nearly unchanged.

**Figure 7:**
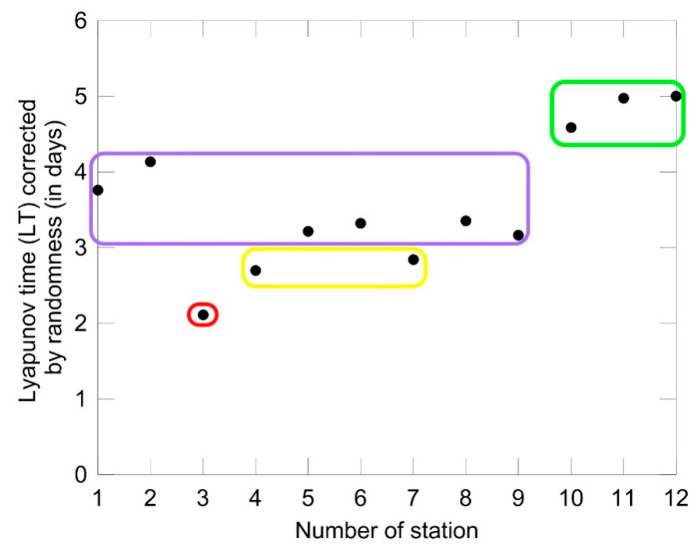
Predictability of the standardized daily discharge data of the Brazos River, given by the Lyapunov time (LT) corrected for randomness (in days).

Figure 7 shows the predictability of the standardized daily discharge data of the Brazos River, given by the Lyapunov time (LT) corrected for randomness (in days). From this figure it is seen that LT corrected for randomness increases from two to five days. Such distribution corresponds to the order of clusters in the 3D scatter plot (Figure 5) along the diagonal, from the upper right corner to the lower left one.

## 5. Conclusions

We compared the average-linkage clustering hierarchical algorithm with three clustering algorithms based on the compression-based dissimilarity measure (NCD), permutation distribution dissimilarity measure (PDDM), and Kolmogorov distance (KD) for daily streamflow discharge data from twelve gauging stations on the Brazos River in Texas (USA), for the period 1989–2016. The algorithm was also compared with K-means clustering based on Kolmogorov complexity (KC), the highest value of Kolmogorov complexity spectrum (KCM), and the largest Lyapunov exponent (LLE). The following conclusions are drawn from this study:

1. We considered the way of selecting suitable information measures for K-means clustering. Accordingly, we selected three measures (i.e., the LLE, KC, and KCM).
2. This choice was made for the following reasons. There are many factors, both natural and human-induced, that cause continuous changes in streamflow time series and; therefore, in its nonlinearity and complexity, of the Brazos River, and its drainage basin. Additionally, because streamflow processes are unavoidably influenced by measurement at gauging stations (including uncertainties in the single determination of river discharge) and dynamical noise that increases LLE under the influence of noise.
3. Using a dissimilarity matrix based on NCD, PDDM, and KD for daily streamflow discharge data from twelve gauging stations, the agglomerative average-linkage hierarchical algorithm was applied. We selected the KD clustering algorithm as the most suitable among others.
4. The dendrogram gave the indication that the gauging stations may be grouped either in three or four clusters. For statistical analysis (3D scatter plot specified by the vectors KC, KCM, and LLE, and calculating the centroids (means) of the clusters), we chose four clusters.
5. On the basis of analysis of variance (ANOVA) results, it could be concluded that there was highly significant differences between mean values of four clusters, which confirmed that the choice of the number of clusters was correctly done.
6. The predictability of standardized daily discharge data of the Brazos River given by the Lyapunov time (LT), corrected for randomness (in days), increased in the following way: (i) three to four days for Cluster 1 (1_08082500, 2_08088000, 5_08090800, 6_08091000, 8_08096500, and 9_08098290 stations); (ii) up to four days for Cluster 2 (3_08088610 station); (iii) approximately three days for Cluster 3 (4_08089000 and 7_08093100 stations); and approximately five days for Cluster 4 (10_08111500, 11_08114000, and 12_08116650 stations).

## Figures and Tables

**Table 1:** Basic descriptive statistics of the daily discharge data of the Brazos River for the period (1989–2016); (the first number indicates the order of the station used in this study).

USGS Code	Station	Mean	Median	Min	Max	IQR	SD*_i_*
1_08082500	Seymour	223.5	51.0	0.0	30,700.0	130.0	907.9
2_08088000	South Bend	613.0	110.0	0.0	43,800.0	320.0	2209.8
3_08088610	Graford	623.5	109.0	4.1	43,800.0	300.0	2306.9
4_08089000	Palo Pinto	723.7	133.0	8.5	39,700.0	361.0	2557.9
5_08090800	Dennis	974.4	195.0	0.0	79,500.0	418.0	3600.3
6_08091000	Glen Rose	1078.8	86.0	1.5	82,100.0	530.0	4093.9
7_08093100	Aquilla	1561.2	445.0	1.2	27,100.0	1118.0	3687.3
8_08096500	Waco	2456.1	695.0	0.5	44,000.0	1775.0	5237.7
9_08098290	Highbank	3103.7	873.5	30.0	70,300.0	2240.0	6148.1
10_08111500	Hempstead	8014.3	2520.0	58.0	137,000.0	7650.0	12,821.1
11_08114000	Richmond	8523.8	2855.0	182.0	102,000.0	8660.0	13,232.0
12_08116650	Rosharon	8851.4	3060.0	27.0	109,000.0	9080.0	13,638.0

**Table 2:** Largest Lyapunov exponent (LLE), Kolmogorov complexity (KC), and the highest value of Kolmogorov complexity spectrum (KCM) of standardized daily discharge data on the Brazos River.

USGS Code	Station	LLE	KC	KCM
1_08082500	Seymour	0.158	0.266	0.489
2_08088000	South Bend	0.038	0.242	0.446
3_08088610	Graford	0.394	0.474	0.682
4_08089000	Palo Pinto	0.032	0.371	0.658
5_08090800	Dennis	0.042	0.311	0.510
6_08091000	Glen Rose	0.051	0.301	0.508
7_08093100	Aquilla	0.055	0.352	0.581
8_08096500	Waco	0.061	0.298	0.526
9_08098290	Highbank	0.061	0.316	0.422
10_08111500	Hempstead	0.027	0.218	0.285
11_08114000	Richmond	0.014	0.201	0.260
12_08116650	Rosharon	0.018	0.200	0.252

**Table 3:** The centroids (means) of the clusters.

Information Measure	Cluster 1	Cluster 2	Cluster 3	Cluster 4
KC	0.289	0.474	0.362	0.206
KCM	0.484	0.682	0.620	0.266
LLE	0.069	0.394	0.044	0.020

**Table 4:** Analysis of variance (ANOVA) table for K-means clustering. The symbols introduced have the following meaning: SS—sum of squares; df—degrees of freedom; F—calculated value of F-test; *P*—value.

Variable	Between SS	df	Within SS	df	F	*P*
KC	0.064679	3	0.004561	8	37.81	0.00005
KCM	0.215958	3	0.011885	8	48.46	0.00002
LLE	0.112645	3	0.010489	8	28.64	0.00013
